# Health-state utility values and their time to deterioration in informal caregivers of older patients with chronic diseases

**DOI:** 10.3389/fpubh.2025.1531608

**Published:** 2025-04-30

**Authors:** Astrid Pozet, Antoine Falcoz, Cécile Roller, Taha Jai, Aurelia Meurisse, Virginie Nerich

**Affiliations:** ^1^CHU de Besançon, Délégation à la Recherche Clinique et à l’Innovation, Besançon, France; ^2^CHU de Besançon, INSERM, EFS-BFC, UMR 1098, Methodological and Quality of Life in Oncology Unit, Besançon, France; ^3^CHU de Besançon, Centre investigation Clinique, Besançon, France; ^4^CHU de Besançon, Pôle Pharmacie, Besançon, France; ^5^Université de Franche-Comté, CHU Besançon, INSERM, EFS-BFC, UMR 1098, Pôle Pharmacie, Besançon, France

**Keywords:** caregiver, health-state utility value, older patient, social worker support, time to deterioration, economic value

## Abstract

**Objectives:**

This study aimed to assess health state utility values (HSUVs) in caregivers of older patients with chronic diseases receiving or not receiving social worker support.

**Methods:**

This multicentric open-label randomized study assigned caregivers to receive either an informational booklet alone or one accompanied by social worker support. Caregivers completed EQ-5D-3L each semester for 24 months. We reported caregiver HSUVs at baseline and after 6, 12, 18, and 24 months using EQ-5D-3L utility index scores and exploring their time to deterioration (TTD).

**Results:**

Among 179 included caregivers, the percentage reporting some or extreme problems on five EQ-5D-3L dimensions remained almost stable over time with a median EQ-5D-3L utility index score of 0.89 [0.80–1.00] at baseline (*n* = 177), 0.80 [0.80–0.89] at M6 (*n* = 125), and 0.80 [0.73–0.91] at M24 (*n* = 81). Among the respondents, 62% (*n* = 109) experienced a deterioration in EQ-5D-3L utility index score, with a median TTD of 9.1 months [95%CI 6.2–14.9] in the control group (CG) and 9.5 months [6.3–14.4] in the supportive intervention group (SIG) (HR = 1.06 [0.73–1.54]), *p*-value = 0.76.

**Conclusion:**

Our study provides a catalog of HSUVs across different caregiver profiles and at various follow-up time points, which can inform future economic evaluations.

**Clinical trial registration:**

ClinicalTrials.gov, NCT02626377.

## Introduction

Informal caregivers’ health-related quality of life (HRQoL) is closely related to the HRQoL of the person they are helping (1, 2). From initial diagnosis to successive phases of stabilization, remission, or progressive decline and palliation, caregiving requires profound changes in the caregiver’s lifestyle, impacting physical, emotional, and social wellbeing. As caregivers navigate these phases, they face practical, organizational, and economic challenges that can significantly affect their HRQoL (3, 4). As part of the overall concept of QoL, monitoring caregiver anxiety, depression symptoms, and burden is of particular importance, and their related scores are useful for detecting early signs of mental health deterioration (5, 6). These scores are used to help caregivers further preserve their wellbeing or prevent health deterioration, which could subsequently jeopardize their ability to care for themselves and the patient. The French multicenter open-label randomized study of *Informal Carers of Older people patients* (ICE study) showed that depression statistically significantly increased over time [1.4 ± 4.0 between baseline and 12 months (*p*-value = 0.01) and 1.7 ± 4.1 between baseline and 24 months (*p*-value = 0.02)] among the 89 informal caregivers of older patients with chronic diseases randomized in the control group (not receiving social support) (7, 8).

Despite the recognized impact of caregiving on HRQoL, there is a lack of studies assessing the economic burden and the long-term consequences of caregiving on informal caregivers of patients with chronic diseases, even though they are essential for rational decision-making in healthcare (9–14). A major challenge in economic evaluation is to provide cost-effectiveness data that are relevant to daily practice and that may optimize the use of healthcare resources. Cost–utility analysis is recommended when HRQoL is identified as an important health effect of the compared interventions (15). Health outcome is measured by the length of life weighted by a valuation of the HRQoL, for instance, via the EuroQOL-5 dimensions instrument (EQ-5D), represented by health-state utility values (HSUVs), to produce quality-adjusted life years (QALYs). The HSUVs range from 1 (best imaginable health state, i.e., perfect health) to 0 (worst imaginable health state, i.e., death) using patient preference-based measures (16, 17).

To our knowledge, no study has specifically evaluated the impact of any intervention on changes in HSUVs over time among informal caregivers. The time-to-HRQoL score deterioration (TTD) approach could provide clinicians with relevant and meaningful data that are more likely to influence clinical decision-making (18).

To address this gap, based on data collected for each informal caregiver included in the ICE study, the primary aim of the present study was to assess their HSUVs at baseline and after 6, 12, 18, and 24 months, depending on whether informal caregivers received social worker support. The secondary aims were to investigate the TTD as well as factors associated with changes in HSUVs.

## Methods

### Design and study population

As previously presented, informal caregivers included in the ICE study were ≥18 years, identified by the patient or self-identified as the primary caregiver, not employed by a healthcare organization, and residing in the French region Burgundy-Franche-Comté (7, 8). The caregivers supported patients aged ≥60 years with neurodegenerative disease (idiopathic Parkinson’s or Alzheimer’s disease), cancer (breast, prostate, or colorectal), age-related macular degeneration, or neurovascular disease (stroke). Informal caregivers of patients living in institutions and caregivers under legal protection were not included. As previously detailed, they were randomly assigned (1:1 ratio) to receive an information booklet and the intervention from a social worker in the supportive intervention group (SIG) or to exclusively receive an information booklet in the control group (CG) (8). Randomization was conducted by the data manager with an interactive web response system, using the minimization technique with stratification according to center, age (80 years or older versus below 80 years), gender, and stage (severity of the disease). Investigators and caregivers were not masked for group allocation. The theoretical framework justifying social worker interventions is to prevent caregiver QoL deterioration and to better support their involvement and communication, thereby contributing to preserving the quality of care provided to the patient. Social worker intervention used the Linear Analogue Scale Assessment (LASA) questionnaire and semi-directive interviews to support the emergence of caregiver needs and specifically address their needs through counseling regarding home services, medical home care, community services (support group), proposing services to promote safety and assist with daily needs (meal delivery, medical alert service), counseling from a psychologist, admission of caregivers for respite care, and encouraging caregivers to take care of themselves and regularly attend consultation with their physician. Interventions from social workers were scheduled at 6, 12, 18, and 24 months (M6, M12, M18, and M24). The inclusion consisted of a 1-h visit to the caregiver’s home, aimed at evaluating the level of difficulties experienced by the caregiver using the LASA questionnaire and assessing caregiver needs. The visit also aimed to detect early signs of burden through a standardized semi-structured interview. The booklets provided access to relevant external assistance structures and support programs and included information regarding local legislation, administrative procedures, daily living management, and potential consequences related to the caregiving role.

Due to low recruitment and despite several amendments, the initially planned sample size was unachievable. Therefore, the final sample size of the ICE study did not allow for the statistical analyses initially planned, particularly lacking the power to directly compare both groups (7).

### Health-state utility value elicitation

HSUVs were elicited using the three-level version of the EuroQOL-5 dimensions instrument (EQ-5D-3L), a validated generic multi-attribute utility instrument (19). This generic preference-based measure is recommended in many jurisdictions, such as France (15). Designed as a self-completion questionnaire to describe HRQoL, it comprises five dimensions: mobility, self-care, usual activities, pain/discomfort, and anxiety/depression. Each of these dimensions can take one of the three response levels reflecting severity: no problems, some problems, and extreme problems. The EuroQOL-5D visual analog scale (EQ VAS) complements the EQ-5D descriptive system and records the respondent’s self-rated health on a vertical scale ranging from 0 (worst imaginable health state) to 100 (best imaginable health state). EQ-5D-3L was self-administered to all informal caregivers according to the predefined schedule in the ICE protocol, at baseline, then every 3 months, and up to 24 months (7).

### Endpoints

The primary endpoint was the EQ-5D-3L utility index scores calculated using a specific French time trade-off-derived value set developed by Chevalier et al. (20). Secondary endpoints included EQ VAS, TTD, and associated factors.

### Statistical analyses

All analyses were performed using a modified intention-to-treat principle, i.e., including all informal caregivers allocated to randomization and randomized with a calculated EQ-5D-3L utility index score at baseline, i.e., at inclusion. They included all data available at each considered follow-up time point.

The number and the percentage of informal caregivers reporting each level of problem on each item of the EQ-5D-3L were described at baseline, M6, M12, M18, and M24 in the overall population (21). The EQ-5D-3L utility index scores and EQ VAS were described by the median and the interquartile range (i.e., the region between the 25th and 75th percentile) [IQR] in the overall population, in each group, i.e., SIG and CG, and for each potential predictive factor, at M6, M12, M18, and M24. Both were also described by the mean and the standard deviation in the overall population. No statistical comparisons of EQ-5D-3L utility index scores between different measurement times were conducted due to the limited sample size.

Derived from the literature, the TTD of the EQ-5D-3L utility index score was defined as the time interval from inclusion to the occurrence of a decrease of at least 0.08 points of the EQ-5D-3L utility index score compared to baseline (22). TTD of EQ VAS was defined as the time interval from inclusion to the occurrence of a first decrease of at least 7 points of the EQ VAS compared to baseline (22). Informal caregivers with no follow-up EQ-5D-3L were censored 1 day after their inclusion. Informal caregivers with no deterioration observed before their drop-out were censored at the time the last EQ-5D-3L was completed. The TTD curves were estimated using the Kaplan–Meier method and described using the median and 95% confidence intervals (CIs).

Baseline parameters collected at inclusion were explored to identify their association with TTD, among which parameters related to patients included gender, age, and disease and caregivers included gender, age, marital status, living situation, relationship with patient, professional situation, and household incomes. The analyses of associated factors of TTD were performed using a 2-step approach. First, the association of potential predictive factors with TTD was examined using the univariate Cox proportional hazards regression model. Second, all variables with a *p*-value of < 0.15 in the univariate analysis and the group parameter was entered in a multivariate model. Quantitative and qualitative variables were transformed, whenever possible, into dichotomic variables using different successive cutoff points. The results of univariate and multivariate analyses are presented with the *hazard ratio* (HR), 95% confidence intervals [95% CIs], and *p*-values.

Mixed models for repeated measures were used, including all time points of up to M24 and including the following effects: randomization group, time, allocation-by-time interaction, adjustment to baseline score, and baseline score-by-time interaction. Random effects on intercept and time were used to reflect individual variations. Adjusted mean changes at M12 and M24 were reported with a 95% confidence interval for EQ-5D-3L utility index score and EQ VAS in each group. Statistically and minimally important differences in clinical significance were indicated.

Statistical analyses were performed using SAS version 9.4 (SAS Institute Inc., Cary, NC, USA). *p*-values of <0.05 were considered statistically significant and provided for exploratory purposes, with all tests being two-sided.

## Results

**Figure 1:**
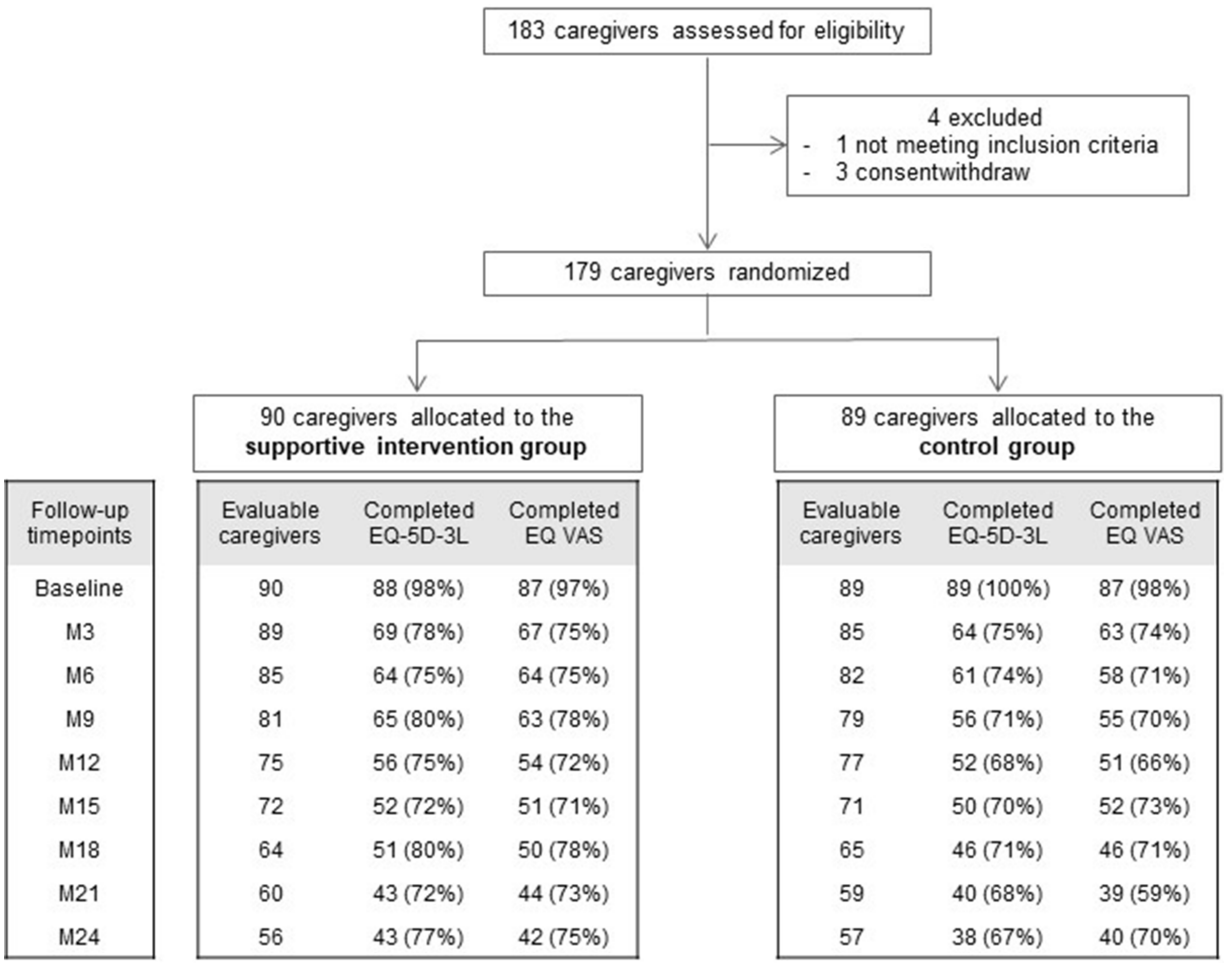
Trial profile (France, 2015–2019). M, month.

From October 2015 to May 2019, 183 caregivers were recruited, and 179 were randomly assigned to the SIG (*n* = 90) or the CG (*n* = 89). Among them, 177 (99%) completed the EQ-5D-3L at baseline, including 177 caregivers (99%) with an EQ-5D-3L utility index score and 174 (97%) with an EQ VAS (Figure 1). The completion rates reached reliable percentages (mainly >70%) at all follow-up time points. In the SIG, the completion rates of EQ-5D-3L were 56 out of 75 (75%) and 43 out of 56 (77%) at M12 and M24, respectively, and in the CG, 52 out of 77 (68%) and 38 out of 57 (67%) at M12 and M24, respectively.

### Health profiles, EQ-5D-3L utility index scores, and EQ VAS

At baseline, few informal caregivers reported some or extreme problems with mobility (*n* = 21, 12%), self-care (*n* = 0, 0%), and usual activities (*n* = 18, 10%) dimensions (Table 1). Almost two thirds (*n* =  113, 64%) and half (*n* = 84, 47%) reported some or extreme problems on the pain/discomfort and anxiety/depression dimensions respectively. The percentage of informal caregivers reporting some or extreme problems on five dimensions has remained almost stable over time: (1) the EQ-5D-3L utility index score with a median of 0.89 [0.80–1.00] at baseline and 0.80 [0.80–0.89] at M6 (*n* = 125) or 0.80 [0.73–0.91] at M24 (*n* = 81), and (2) EQ VAS with a median of 75 [60–85] at baseline and 75 [60–85] at M6 (*n* = 122) or 70 [70–80] at M24 (*n* = 82). The EQ-5D-3L utility index score also remained almost stable over time regardless of all parameters collected at baseline (Table 2).

**Table 1:** Number and percentage of caregivers’ responses to the EQ-5D-3L and EQ-5D-3L utility score and EQ VAS mean and median at each time-point of assessment (France, 2015–2019).

	Baseline	M6	M12	M18	M24
EQ-5D-3L					
Mobility					
Level 1: no problem	156 (88)	111 (89)	91 (84)	83 (86)	70 (86)
Level 2: some problems	21 (12)	14 (11)	17 (16)	14 (14)	11 (14)
Level 3: extreme problems	0 (0)	0 (0)	0 (0)	0 (0)	0 (0)
Total	177 (100)	125 (100)	108 (100)	97 (100)	81 (100)
Self-care					
Level 1: no problem	177 (100)	123 (98)	106 (98)	92 (95)	81 (100)
Level 2: some problems	0 (0)	2 (2)	2 (2)	4 (4)	0 (0)
Level 3: extreme problems	0 (0)	0 (0)	0 (0)	1 (1)	0 (0)
Total	177 (100)	125 (100)	108 (100)	97 (100)	81 (100)
Usual activities					
Level 1: no problem	159 (90)	106 (85)	87 (81)	78 (80)	68 (84)
Level 2: some problems	17 (9)	18 (14)	21 (19)	19 (20)	13 (16)
Level 3: extreme problems	1 (1)	1 (2)	0 (0)	0 (0)	0 (0)
Total	177 (100)	125 (100)	108 (100)	97 (100)	81 (100)
Pain/discomfort					
Level 1: no problem	64 (36)	32 (26)	29 (27)	23 (24)	24 (30)
Level 2: some problems	107 (61)	90 (72)	78 (72)	71 (73)	55 (68)
Level 3: extreme problems	6 (3)	3 (2)	1 (1)	3 (3)	2 (2)
Total	177 (100)	125 (100)	108 (100)	97 (100)	81 (100)
Anxiety/depression					
Level 1: no problem	93 (53)	53 (42)	44 (41)	42 (43)	36 (44)
Level 2: some problems	73 (41)	63 (50)	48 (44)	50 (52)	39 (48)
Level 3: extreme problems	11 (6)	9 (7)	16 (15)	5 (5)	6 (7)
Total	177 (100)	125 (100)	108 (100)	97 (100)	81 (100)
EQ-5D-3L utility index score					
Mean ± standard deviation	0.82 ± 0.18	0.79 ± 0.19	0.76 ± 0.21	0.77 ± 0.20	0.80 ± 0.18
Median [IQR]	0.89 [0.80–1.00]	0.80 [0.80–0.89]	0.80 [0.64–0.89]	0.80 [0.73–0.89]	0.80 [0.73–0.91]
EQ VAS					
Number	174 (100.0)	122 (100.0)	105 (100.0)	96 (100.0)	82 (100.0)
Mean ± standard deviation	72.8 ± 17.0	71.7 ± 16.4	69.9 ± 16.3	69.8 ± 17.0	70.0 ± 16.2
Median [IQR]	75.0 [60.0–85.0]	75.0 [60.0–85.0]	71.0 [60.0–80.0]	70.0 [60.0–80.0]	70.0 [60.0–80.0]

IQR, interquartile range; M, month.

**Table 2:** EQ-5D-3L utility index score at baseline and 6, 12, 18, and 24 months (France, 2015–2019).

	Baseline			M6			M12			M18			M24		
	*n*	Median	IQR	*n*	Median	IQR	*n*	Median	IQR	*n*	Median	IQR	*n*	Median	IQR
Overall population	177	0.89	0.80–1.00	125	0.80	0.80–0.89	108	0.80	0.64–0.89	97	0.80	0.73–0.89	81	0.80	0.73–0.91
Randomization group															
Supportive intervention	88	0.89	0.80–0.91	64	0.80	0.78–0.89	56	0.80	0.69–0.89	51	0.80	0.64–0.89	43	0.80	0.64–0.91
Control	89	0.89	0.80–1.00	61	0.80	0.80–0.89	52	0.80	0.63–0.91	46	0.80	0.73–0.89	38	0.89	0.73–0.91
Patient sex															
Male	79	0.89	0.80–0.91	57	0.80	0.80–0.89	57	0.80	0.63–0.89	45	0.80	0.73–0.91	37	0.80	0.73–0.91
Female	98	0.89	0.80–1.00	68	0.80	0.80–0.89	51	0.80	0.64–0.89	52	0.80	0.69–0.89	44	0.89	0.69–0.90
Patient age, years															
<80	123	0.89	0.80–1.00	87	0.80	0.80–0.89	81	0.80	0.64–0.89	67	0.80	0.64–0.89	59	0.80	0.64–0.91
≥80	54	0.84	0.80–0.91	38	0.80	0.75–0.89	27	0.80	0.64–0.89	30	0.80	0.80–0.89	22	0.80	0.80–0.89
Patient disease															
Neurodegenerative disease	66	0.80	0.80–0.89	44	0.80	0.74–0.89	36	0.80	0.63–0.89	34	0.80	0.73–0.89	31	0.80	0.64–0.89
Stroke/age-related macular degeneration	21	0.91	0.89–1.00	14	0.89	0.58–0.91	12	0.89	0.84–0.91	14	0.84	0.73–0.89	11	0.89	0.73–0.89
Cancer	90	0.89	0.80–1.00	67	0.80	0.80–1.00	60	0.87	0.64–0.91	49	0.80	0.73–0.91	39	0.89	0.64–1.00
Patient neurodegenerative disease															
Yes	66	0.80	0.80–0.89	44	0.80	0.74–0.89	36	0.80	0.63–0.89	34	0.80	0.73–0.89	31	0.80	0.64–0.89
No	111	0.89	0.80–1.00	81	0.89	0.80–0.91	72	0.89	0.69–0.91	63	0.80	0.73–0.91	50	0.89	0.73–1.00
Caregiver sex															
Female	120	0.89	0.80–0.91	85	0.80	0.80–0.89	80	0.80	0.63–0.89	67	0.80	0.73–0.91	51	0.80	0.64–0.91
Male	57	0.89	0.73–1.00	40	0.84	0.77–0.89	28	0.89	0.64–0.89	30	0.80	0.64–0.89	30	0.89	0.80–0.91
Caregiver age, years															
<65	82	0.89	0.80–1.00	52	0.80	0.80–0.89	52	0.82	0.64–0.91	45	0.80	0.73–0.91	33	0.89	0.64–1.00
≥65	95	0.89	0.73–0.91	73	0.80	0.75–0.89	56	0.80	0.64–0.89	52	0.80	0.73–0.89	48	0.80	0.73–0.89
Marital status, living situation															
Married, common-law couple, civil partnerships	149	0.89	0.80–0.91	103	0.80	0.80–0.89	89	0.80	0.64–0.89	82	0.80	0.73–0.89	70	0.80	0.73–0.91
Other (single, separated, divorced, or widowed)	28	0.88	0.80–1.00	22	0.80	0.80–0.89	19	0.80	0.49–0.91	15	0.80	0.80–0.91	11	0.89	0.64–1.00
Caregiver–patient relationship (caregiver taking care of his/her)															
Spouse	117	0.89	0.80–0.91	86	0.80	0.80–0.89	73	0.80	0.64–0.89	64	0.80	0.73–0.89	57	0.80	0.73–0.89
Mother/Father	41	0.89	0.80–1.00	29	0.80	0.80–0.89	24	0.80	0.64–0.90	26	0.82	0.80–0.91	17	0.80	0.64–1.00
Other (friend, neighbor, sister, etc.)	19	0.91	0.89–1.00	10	0.84	0.80–0.89	11	0.89	0.64–1.00	7	0.80	0.64–0.91	7	0.89	0.51–0.91
Professional situation															
Professional activity	41	0.89	0.80–1.00	27	0.80	0.80–1.00	25	0.84	0.80–0.91	26	0.80	0.80–0.91	20	0.84	0.73–1.00
Retired	122	0.89	0.80–0.91	88	0.80	0.78–0.89	74	0.80	0.64–0.89	65	0.80	0.73–0.89	57	0.89	0.73–0.91
Other: sick leave, unemployment, job training, etc.	14	0.80	0.58–0.89	10	0.80	0.51–0.80	9	0.64	0.51–0.80	6	0.71	0.37–0.80	4	0.56	0.49–0.72
Household incomes, euros/month															
< 800	6	0.80	0.64–0.89	4	0.58	0.26–0.84	3	0.80	0.15–0.91	3	1.80	0.36–0.80	3	0.80	0.64–0.94
From 800 to 1,500	16	0.89	0.65–0.91	14	0.84	0.80–0.91	11	0.64	0.49–0.89	7	0.80	0.64–0.80	6	0.77	0.49–0.89
From 1,501 to 3,000	86	0.89	0.80–0.91	62	0.80	0.80–0.89	52	0.80	0.64–0.89	54	0.80	0.80–0.89	40	0.89	0.77–0.91
Up to 3,001	50	0.89	0.73–1.00	33	080	0.75–0.89	30	0.89	0.80–1.00	24	0.80	0.73–0.89	20	0.84	0.77–0.96

IQR, interquartile range; M, month; *n*, number.

### TTD of EQ-5D-3L utility index score and EQ VAS

**Figure 2:**
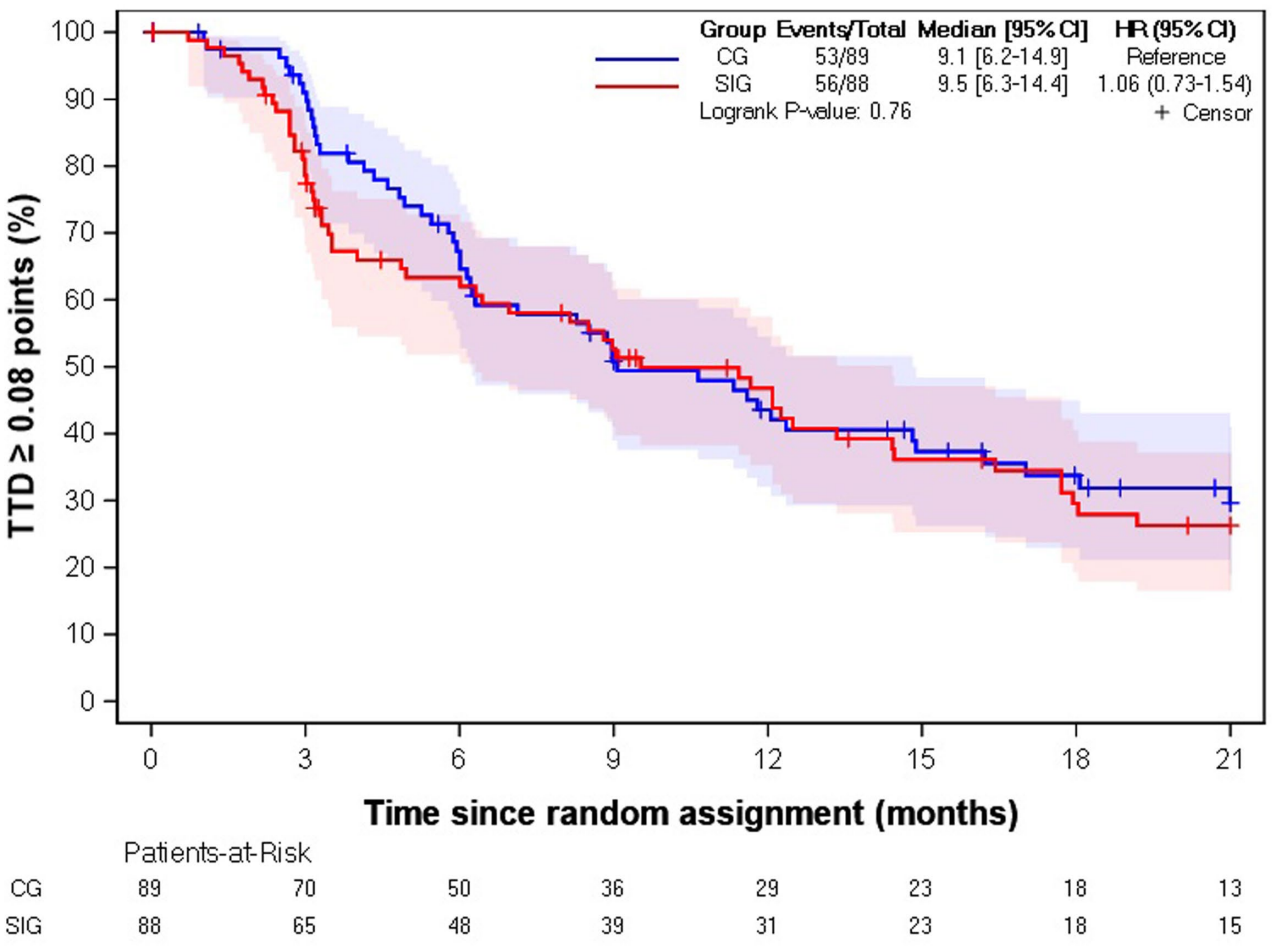
Kaplan–Meier estimation of time to deterioration of EQ-5D-3L utility index score. CG, control group; SIG, supportive intervention group; HR, hazard ratio; CI, confidence interval; TTD, time to deterioration.

Among the 177 informal caregivers with EQ-5D-3L utility index scores at baseline, 62% (*n* = 109) experienced a deterioration in EQ-5D-3L utility index score with a median TTD of 9.1 months [6.2–14.9] in the CG and 9.5 months [6.3–14.4] in the SIG (HR = 1.06 [0.73–1.54], *p*-value = 0.76) (Table 3; Figure 2). In the univariate and multivariate analyses, the only parameter significantly associated with longer TTD was the caregiver’s professional situation (*p*-value = 0.08).

**Table 3:** Time to deterioration of EQ-5D-3L utility index score (France, 2015–2019).

	Time to deterioration, months			Univariate analysis			Multivariate analysis		
	*n* (%)	Median	95% CI	Hazard ratio	95% CI	*p*-value	Hazard ratio	95% CI	*p*-value
Randomization group						0.76			0.74
Control	53 (59.6)	9.1	6.2–14.9	1			1		
Supportive intervention	56 (63.6)	9.5	6.3–14.4	1.06	0.73–1.54		1.07	0.73–1.55	
Patient sex						0.65			
Female	56 (57.1)	9.1	6.0–12.5	1					
Male	53 (67.1)	11.6	6.2–14.9	1.09	0.75–1.59				
Patient age, years						0.65			
<80	76 (61.8)	10.6	6.3–14.5	1					
≥80	33 (61.1)	9.1	5.3–13.3	1.10	0.73–1.66				
Patient disease						0.58			
Cancer	55 (61.1)	11.3	8.3–14.8	1					
Stroke/Age-related macular degeneration	15 (71.4)	9.5	3.1–17.0	1.33	0.75–2.35	0.33			
Neurodegenerative disease	39 (59.1)	9.0	4.9–14.4	1.15	0.77–1.74	0.49			
Patient neurodegenerative disease						0.66			
No	70 (63.1)	11.3	8.1–14.5	1					
Yes	39 (59.1)	9.0	4.9–14.4	1.09	0.74–1.62				
Caregiver sex						0.35			
Male	31 (54.4)	10.6	5.9–23.4	1					
Female	78 (65.0)	9.0	6.4–12.4	1.22	0.80–1.85				
Caregiver age, years						0.84			
<65	50 (61.0)	11.7	7.1–16.2	1					
≥65	59 (62.1)	9.0	6.0–14.4	1.04	0.71–1.52				
Caregiver’s marital status, living situation						0.38			
Married, common-law couple, civil partnerships	90 (60.4)	10.6	7.0–14.5	1					
Other (single, separated, divorced, or widowed)	19 (67.9)	9.5	5.0–12.5	1.25	0.76–2.05				
Caregiver–patient relationship (caregiver taking care of his/her)						0.90			
Spouse	72 (61.5)	10.6	6.4–14.8	1					
Mother/Father	25 (61.0)	8.5	4.3–17.7	1.09	0.69–1.72	0.70			
Other (friend, neighbor, sister, etc.)	12 (63.2)	11.4	5.0–19.2	1.11	0.60–2.05	0.74			
Caregiver professional situation						0.08			0.08
Retired	73 (59.8)	10.6	7.0–14.5	1			1		
Other: sick leave, unemployment, job training, etc.	11 (78.6)	5.8	1.9–9.0	2.02	1.07–3.82	0.03	2.02	1.07–3.82	0.03
Professional activity	25 (61.0)	11.8	5.3–17.7	0.97	0.62–1.53	0.89	0.97	0.62–1.53	0.89
Caregiver household incomes, euros/month						0.82			
< 800	2 (33.3)	NE	2.5-NE	1					
From 800 to 1,500	11 (68.8)	12.1	4.1–18.1	1.24	0.28–5.62	0.78			
From 1,501 to 3,000	57 (66.3)	9.0	6.2–11.7	1.41	0.34–5.77	0.63			
Up to 3,001	28 (56.0)	9.0	5.5–21.0	1.15	0.27–4.84	0.85			

AMD, age-related macular degeneration; CI, confidence interval; *n*, number; NE, not estimable.

**Figure 3:**
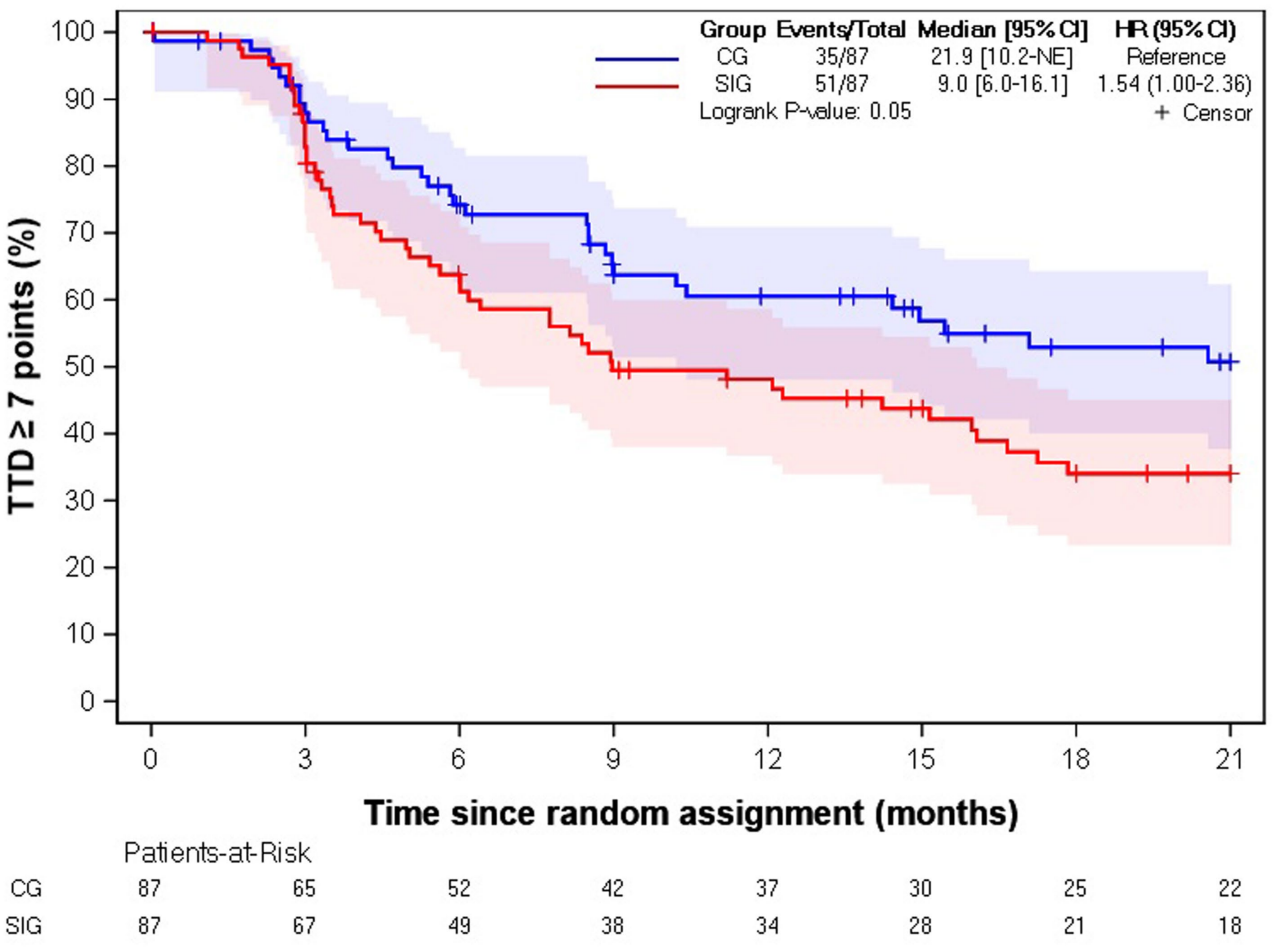
Kaplan–Meier estimation of time to deterioration of EQ VAS score. CG, control group; SIG, supportive intervention group; HR, hazard ratio; CI, confidence interval; TTD, time to deterioration.

Among the 174 informal caregivers with an EQ VAS at baseline, 49% (*n* = 86) experienced a deterioration in EQ VAS with a median TTD of 9.0 months [6.0–16.1] in the SIG and 21.9 months [10.2-not estimable] in the CG (HR = 1.54 [1.00–2.36], *p*-value = 0.05) (Figure 3).

### Caregivers’ adjusted mean change over time in EQ-5D-3L utility index score and EQ VAS

The mixed models for repeated measures did not show clinically significant differences at M12 or M24 for EQ-5D-3L utility index score and EQ VAS (Supplementary material S1).

## Discussion

The present analysis of 177 caregivers with EQ-5D-3L utility index scores at baseline showed that the percentage of informal caregivers reporting some or extreme problems on five dimensions remained stable over time with an EQ-5D-3L utility index score median of 0.89 at baseline and 0.80 as of M6 to M24 regardless of the randomization group. There was no TTD-relevant difference between randomization groups (9.1 months [6.2–14.9] in the CG and 9.5 months [6.3–14.4] in the SIG (HR = 1.06 [0.73–1.54], *p*-value = 0.76)). However, these results must take into account the specificity of the intervention specially designed for the study (8). Indeed, social workers indicated that their role was primarily one of listening and pointed out that caregivers had not taken full advantage of all the opportunities available to them. Several assumptions may explain these missed opportunities. The protocol scheduled semester visits that were inconsistent with current social worker practice, which generally recommends more frequent visits. This finding may have contributed to limiting the ability of social workers to provide appropriate and timely support. More frequent visits, organized at the request of caregivers, would have facilitated the timely and efficient identification and addressing of these needs. In addition, the global interpretation of changes in EQ-5D-3L utility index scores or EQ VAS should be approached with caution due to attrition biases. Indeed, caregivers who discontinued the study early were the most at risk for limited HRQoL, with poor physical dimensions and high burden at baseline, particularly among caregivers withdrawn at M12 (8).

Understanding how HRQoL is impacted by different factors is essential to fully describe the burden of disease on patients and caregivers. For explanatory purposes, our study identified only caregivers’ professional situation factor as an independent predictor of TTD (*p*-value = 0.03). Caregivers on sick leave, unemployed, or in job training had a significantly longer TTD compared to retired caregivers (who are typically older), or those with professional activity (who tend to be more active both professionally and personally). Therefore, these results must be interpreted with caution, considering the low recruitment rate, the limited intervention uptake, the significant heterogeneity of this population, and potential biases. These limitations likely affected the statistical power, reducing the ability to detect significant differences in other variables (sex or age of the caregiver, for instance). Furthermore, potential biases, such as non-response bias, may have influenced the results by affecting the representativeness of the sample. This finding suggests that the lack of observed effects can be attributed not only to the intervention and its ineffectiveness but also to the constraints of the method. However, we can assume that the availability of these caregivers could be their common feature.

It is important to note that several factors have been identified as influencing caregiver experiences in various cultural contexts (23, 24). While not assessed in our study, future research should explore their potential role in caregiver burden.

Thus, our study provides a comprehensible catalog of utility value data per health state for different caregiver profiles and at various follow-up time points. These HSUV data can be used in future health economic modeling. Over the past 20 years, interest in the economic evaluation of healthcare interventions has risen. The results of these evaluations have become increasingly important as criteria for the allocation of healthcare resources. Cost–utility analysis is recommended, for instance, by the Canadian Agency for Drugs and Technologies in Health (CADTH), the French National Authority for Health (HAS), and the National Institute for Health and Care Excellence (NICE) in the United Kingdom when HRQoL is identified as a relevant health effect of the interventions studied and compared, particularly for chronic diseases, such as neurodegenerative disease, stroke, age-related macular degeneration, or cancer (15, 25, 26). However, the variability in the quality of conduct and reporting in health economic evaluations has been well documented since 2000, particularly concerning HSUVs, which are most often derived from the literature or missing altogether (27). Thus, our HSUV catalog will contribute to the development of future caregiver-related cost–utility analyses, an area that is currently not widespread.

## Conclusion

The present analysis of 179 caregivers showed no clinically relevant changes in EQ-5D-3L utility index score and EQ VAS, regardless of the allocation group, in line with the analysis of Pozet et al. (8). However, it provides an HSUV catalog across different caregiver profiles and at various follow-up time points, which can inform future economic evaluations.

## Data Availability

The raw data supporting the conclusions of this article will be made available by the authors, without undue reservation.

## References

[1] Kaschowitz J Brandt M. Health effects of informal caregiving across Europe: a longitudinal approach. Soc Sci Med. (2017) 173:72–80. doi: 10.1016/j.socscimed.2016.11.036, PMID: 27930918

[2] Le DD Ibuka Y. Understanding the effects of informal caregiving on health and well-being: heterogeneity and mechanisms. Soc Sci Med. (2023) 317:115630. doi: 10.1016/j.socscimed.2022.115630, PMID: 36580861

[3] Schofield D Shrestha RN Zeppel MJB Cunich MM Tanton R Veerman JL. Economic costs of informal care for people with chronic diseases in the community: lost income, extra welfare payments, and reduced taxes in Australia in 2015-2030. Health Soc Care Community. (2019) 27:493–501. doi: 10.1111/hsc.12670, PMID: 30378213

[4] BVA, Fondation April. BVA Xsight. (2022). Baromètre des aidants – Avancées et perspectives. Available online at: https://www.bva-xsight.com/sondages/barometre-aidants-bva-fondation-april-avancees-perspectives/

[5] de Oliveira GR Neto JF de Camargo SM Lucchetti ALG Espinha DCM Lucchetti G. Caregiving across the lifespan: comparing caregiver burden, mental health, and quality of life. Psychogeriatrics. (2015) 15:123–32. doi: 10.1111/psyg.12087, PMID: 25521215

[6] Del-Pino-Casado R Rodríguez Cardosa M López-Martínez C Orgeta V. The association between subjective caregiver burden and depressive symptoms in carers of older relatives: a systematic review and meta-analysis. PLoS One. (2019) 14:e0217648. doi: 10.1371/journal.pone.0217648, PMID: 31141556 PMC6541277

[7] Pozet A Lejeune C Bonnet M Dabakuyo S Dion M Fagnoni P. Evaluation of efficacy and efficiency of a pragmatic intervention by a social worker to support informal caregivers of elderly patients (the ICE study): study protocol for a randomized controlled trial. Trials. (2016) 17:531. doi: 10.1186/s13063-016-1622-8, PMID: 27881145 PMC5122007

[8] Pozet A Darnis S Bonnet M Meurisse A Dabakuyo-Yonli TS Lejeune C. Quality of life and needs in caregivers: results from the prospective multicentric open-label randomized study of informal caregivers of elderly patients. Int J Public Health. (2023) 68:1605459. doi: 10.3389/ijph.2023.1605459, PMID: 37711159 PMC10498993

[9] Cagle JG Carr DC Hong S Zimmerman S. Financial burden among US households affected by cancer at the end of life. Psychooncology. (2016) 25:919–26. doi: 10.1002/pon.3933, PMID: 26282448 PMC4956573

[10] Deb A Thornton JD Sambamoorthi U Innes K. Direct and indirect cost of managing Alzheimer’s disease and related dementias in the United States. Expert Rev Pharmacoecon Outcomes Res. (2017) 17:189–202. doi: 10.1080/14737167.2017.1313118, PMID: 28351177 PMC5494694

[11] Bradley CJ. Economic burden associated with cancer caregiving. Semin Oncol Nurs. (2019) 35:333–6. doi: 10.1016/j.soncn.2019.06.003, PMID: 31229344 PMC6660380

[12] Ohno S Chen Y Sakamaki H Matsumaru N Tsukamoto K. Humanistic and economic burden among caregivers of patients with cancer in Japan. J Med Econ. (2020) 23:17–27. doi: 10.1080/13696998.2019.1675672, PMID: 31578893

[13] Wong W. Economic burden of Alzheimer disease and managed care considerations. Am J Manag Care. (2020) 26:S177–83. doi: 10.37765/ajmc.2020.88482, PMID: 32840331

[14] Salsman JM Danhauer SC Moore JB Ip EH McLouth LE Nightingale CL. Systematic review of financial burden assessment in cancer: evaluation of measures and utility among adolescents and young adults and caregivers. Cancer. (2021) 127:1739–48. doi: 10.1002/cncr.33559, PMID: 33849081 PMC8113116

[15] Haute Autorité de Santé. (2025). Choices in methods for economic evaluation. Available online at: https://www.has-sante.fr/jcms/r_1499251/en/choices-in-methods-for-economic-evaluation

[16] Lidgren M Wilking N Jönsson B Rehnberg C. Health related quality of life in different states of breast cancer. Qual Life Res. (2007) 16:1073–81. doi: 10.1007/s11136-007-9202-8. PMID: 17468943

[17] Herdman M Gudex C Lloyd A Janssen M Kind P Parkin D. Development and preliminary testing of the new five-level version of EQ-5D (EQ-5D-5L). Qual Life Res. (2011) 20:1727–36. doi: 10.1007/s11136-011-9903-x, PMID: 21479777 PMC3220807

[18] Anota A Hamidou Z Paget-Bailly S Chibaudel B Bascoul-Mollevi C Auquier P. Time to health-related quality of life score deterioration as a modality of longitudinal analysis for health-related quality of life studies in oncology: do we need RECIST for quality of life to achieve standardization? Qual Life Res. (2015) 24:5–18. doi: 10.1007/s11136-013-0583-6, PMID: 24277234 PMC4282717

[19] Brooks R. EuroQol: the current state of play. Health Policy. (1996) 37:53–72. doi: 10.1016/0168-8510(96)00822-6, PMID: 10158943

[20] Chevalier J de Pouvourville G. Valuing EQ-5D using time trade-off in France. Eur J Health Econ. (2013) 14:57–66. doi: 10.1007/s10198-011-0351-x, PMID: 21935715

[21] EQ-5D User Guides. (2024). Basic information on how to use the EQ-5D-3L instrument. Available online at: https://euroqol.org/publications/user-guides/

[22] Pickard AS Neary MP Cella D. Estimation of minimally important differences in EQ-5D utility and VAS scores in cancer. Health Qual Life Outcomes. (2007) 5:70. doi: 10.1186/1477-7525-5-70, PMID: 18154669 PMC2248572

[23] Hazzan AA Shannon H Ploeg J Raina P Oremus M. Association between caregiver quality of life and the care provided to persons with Alzheimer’s disease: systematic review. Adv Alzheimer’s Dis. (2014) 3:44–53. doi: 10.4236/aad.2014.31006 PMC3610300 23497507

[24] Giannouli V Tsolaki M. What biological factors, social determinants, and psychological and Behavioral symptoms of patients with mild Alzheimer’s disease correlate with caregiver estimations of financial capacity? Bringing biases against older women into focus. J Alzheimer’s Dis Rep. (2022) 6:503–7. doi: 10.3233/ADR-220037, PMID: 36186725 PMC9484131

[25] CADTH. (2025). Canadian Agency for Drugs and Technologies in Health (CADTH). Available online at: https://www.cadth.ca/

[26] NICE. (n.d.) National Institute for health and care excellence (NICE). Available online at: http://publications.nice.org.uk/pmg9 (Accepted 04 April 2013).

[27] Neumann PJ Stone PW Chapman RH Sandberg EA Bell CM. The quality of reporting in published cost-utility analyses, 1976-1997. Ann Intern Med. (2000) 132:964–72. doi: 10.7326/0003-4819-132-12-200006200-00007, PMID: 10858180

