# Supplementary material for: Health-state utility values and their time to deterioration in informal caregivers of older patients with chronic diseases
Source: Front Public Health. 2025 Apr 30;13:1531608. doi: 10.3389/fpubh.2025.1531608 (PMC12074913; doi:10.3389/fpubh.2025.1531608)
Supplement: Supplementary file 1 [file Table_1.docx]

**SUPPLEMENTARY MATERIAL**

**Supplementary Material S1:** Caregivers adjusted mean change over time (mixed model for repeated measures) in EQ-5D-3L utility index score and EQ VAS in each randomization group (France, 2015-2019).

|  | Supportive intervention group | | Control group | |
| --- | --- | --- | --- | --- |
|  | Adjusted mean change | 95% CI | Adjusted mean change | 95% CI |
| EQ-5D-3L utility index score | -0.03 | [-0.06 to -0.01] | -0.06 | [-0.08 to -0.03] |
| M12 | -0.05 | [-0.09 to -0.01] | -0.06 | [-0.10 to -0.02] |
| M24 | -0.04 | [-0.08 to +0.00] | -0.07 | [-0.11 to -0.02] |
| EQ-VAS | -2.7 | [-4.9 to -0.4] | -2.8 | [-5.2 to -0.5] |
| M12 | -2.9 | [-6.2 to 0.4] | -3.6 | [-7.0 to -0.1] |
| M24 | -4.7 | [-8.4 to -1.1] | -2.8 | [-6.5 to 0.9] |

Abbreviation: M = month

Note: Minimal important difference was fixed at 0.08 points for EQ-5D-3L utility index score and 7 points for EQ-VAS score.

Mixed models for repeated measures used for longitudinal analysis included all timepoints until M24 and included the following effects: randomization group, time, allocation-by-time interaction, adjusted on baseline score, and baseline score-by-time interaction.
